# Comparison of Feature Extraction Methods for Physiological Signals for Heat-Based Pain Recognition

**DOI:** 10.3390/s21144838

**Published:** 2021-07-15

**Authors:** Philip Gouverneur, Frédéric Li, Wacław M. Adamczyk, Tibor M. Szikszay, Kerstin Luedtke, Marcin Grzegorzek

**Affiliations:** 1 Institute of Medical Informatics, University of Lübeck, Ratzeburger Allee 160, 23562 Lübeck, Germany; li@imi.uni-luebeck.de (F.L.); grzegorzek@imi.uni-luebeck.de (M.G.); 2 Institute of Health Sciences, Department of Physiotherapy, Pain and Exercise Research Lübeck (P.E.R.L.), University of Lübeck, 23562 Lübeck, Germany; waclaw.adamczyk@uni-luebeck.de (W.M.A.); tibor.szikszay@uni-luebeck.de (T.M.S.); kerstin.luedtke@uni-luebeck.de (K.L.); 3 Laboratory of Pain Research, Institute of Physiotherapy and Health Sciences, The Jerzy Kukuczka Academy of Physical Education, 40-959 Katowice, Poland

**Keywords:** pain recognition, machine learning, deep learning, physiological signals, pain perception

## Abstract

While even the most common definition of pain is under debate, pain assessment has remained the same for decades. But the paramount importance of precise pain management for successful healthcare has encouraged initiatives to improve the way pain is assessed. Recent approaches have proposed automatic pain evaluation systems using machine learning models trained with data coming from behavioural or physiological sensors. Although yielding promising results, machine learning studies for sensor-based pain recognition remain scattered and not necessarily easy to compare to each other. In particular, the important process of extracting features is usually optimised towards specific datasets. We thus introduce a comparison of feature extraction methods for pain recognition based on physiological sensors in this paper. In addition, the PainMonit Database (PMDB), a new dataset including both objective and subjective annotations for heat-induced pain in 52 subjects, is introduced. In total, five different approaches including techniques based on feature engineering and feature learning with deep learning are evaluated on the BioVid and PMDB datasets. Our studies highlight the following insights: (1) Simple feature engineering approaches can still compete with deep learning approaches in terms of performance. (2) More complex deep learning architectures do not yield better performance compared to simpler ones. (3) Subjective self-reports by subjects can be used instead of objective temperature-based annotations to build a robust pain recognition system.

## 1. Introduction

Pain can indicate health problems of various kind and serves as natural protective mechanism against harm. It is especially important in medicine, as it comprises both symptom and disease [1]. One of the most common definitions of pain dates back to 1979 and is defined by the International Association for the Study of Pain (IASP) as “an unpleasant sensory and emotional experience associated with actual or potential tissue damage, or described in terms of such damage” [2]. While even this fundamental specification is under debate for revision [3,4,5], pain assessment has remained the same for decades, despite the fact that precise pain management is essential in successful health care. As a matter of fact, pain assessment is not ideal and treatment remains sub-optimal quite often [6,7,8]. The current gold standard for pain assessment consists of self-report [9]. Here the subjective impression of the patient is communicated and shared with clinicians. Often, pain is verbally rated on a Numerical Rating Scale (NRS), where 0 is “no pain” and 10 corresponds to “worst pain imaginable” or located on a Visual Analogue Scale (VAS), a visual line with verbal anchors “no pain” to “worst imaginable pain” at either end [10]. Although being the current gold standard, self-reports come along with major drawbacks in clinical practice [11]. With pain being “a highly individual experience” ([12], p. 5), self-reports constitute a subjective snapshot of patients’ level of pain, resulting in mainly four disadvantages. (1) *Subjectivity*: The comparability of self-reports is complicated by the fact that pain represents a subjective experience. Individual differences in pain result from expectation and empirical knowledge and are additionally influenced by genetic and sociological factors [13]. Thus, outcomes of self-reports have a large variance across individuals. (2) *Consciousness*: To be able to communicate pain, subjects need to be capable of expressing their experience. Children and patients affected by certain diseases, for example, Alzheimer, may have difficulty communicating their concerns explicitly. In addition, patients need to be conscious which is not the case in all medical settings (e.g., coma patients). (3) *Expenditure of time*: Retrieving a pain estimation via self-reports is time-consuming for medical staff. A continuous measurement is nearly impossible in real life. (4) *Validity*: It is questioned whether simple one-dimensional ratings of pain can describe the symptom accurately enough, as they greatly simplify its complexity [14,15]. Newer concepts using graphical faces with different pain expressions for children [16] and more complex questionnaires, like the *McGill Pain Questionnaire* [17] and a nociception coma scale [18] have been introduced but cannot overcome the aforementioned problems nor did they enhance the current gold standard. Especially the issue of nonverbal patients unable to self-report led to clinical practice recommendations to evaluate pain [19].

**Figure 1:**
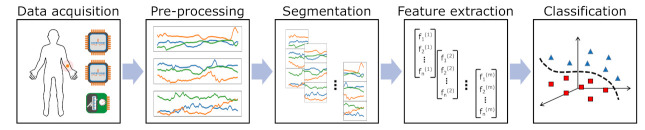
Pattern recognition chain including several steps that should be optimised in parallel to yield the best performance.

These observations underline the clear need for an objective, continuous and automatised pain measurement to improve health care and support medical staff. Recent attempts tried to address this issue by applying *machine learning approaches* on recorded and annotated pain data. Several approaches on how to induce pain to acquire such data have been introduced, ranging from physical exercises for patients with shoulder pain to various stimulus types. While being different from clinical pain, heat-induced pain using a thermode has become dominant as it is one of the most commonly used stimuli to induce experimental pain (for example, 10 years of laboratory research include 34 different studies emphasising heat pain [20] out of 105 various studies on pain) and it is simple to perform. Moreover, it was shown that heat-induced pain is comparable to clinical pain, for example, sharing common mechanisms with postoperative pain [21] and “that the same factors shaping response to experimental pain stimuli also contribute to the experience of clinical pain” [22]. Pain recognition from the machine learning perspective is usually translated either into a classification problem where each class represents a certain level of pain (e.g., no pain, low, or high pain) or into a regression problem where models estimate pain intensity. Typically, the implementation of such classification systems follows the Pattern Recognition Chain, a standardised procedure illustrated in Figure 1, that includes the following steps: (1) *Data acquisition*: Starting by acquiring a dataset of the phenomena to be recognised for training and evaluation purposes. (2) *Pre-processing*: Data is then often pre-processed to reduce noise and remove artefacts. (3) *Segmentation*: Afterwards, the initial data records are segmented into shorter data segments. (4) *Feature extraction*: Often, classifiers are trained on *features* instead of the raw data. These *features* try to outline a dense but still informative representation of the input. (5) *Classification*: The sequence is finalised by training a classification model on the retrieved characteristics that is evaluated at the end. While all the distinct steps contribute to the performance of the systems, especially the data acquisition and feature extraction remain crucial to build a successful classification model. Being the first step, poor data collection can form a bottleneck in the chain that later parts suffer from. Especially, small datasets make it difficult to create a generalising classification model that would perform sufficiently on unseen data and training data including outliers and artefacts further complicate the classification task. Moreover, as classifiers are trained based on features instead of the raw data, the transformation in-between is particularly important. Wrongly engineered features can distract classifiers from the actual task, while proper ones comprise the underlying information yet greatly reducing the dimensionality of the data.

Recent studies on automated pain recognition have proposed their own approaches on the existing benchmark datasets (in particular BioVid Heat Pain Database (BVDB)) but remain scattered and difficult to compare because of divergent evaluation frameworks. Besides various pre-processing steps, varying segmentation techniques, and mixed classification models have been introduced which make it complicated to account for the best feature extraction method. Thus, we introduce an experimental setup allowing a fair comparison of feature extraction approaches, that we used on several datasets with different labelling strategies. The pattern recognition chain was kept identical to capture the influence of several feature extraction approaches on the evaluation results. In detail, pre-processing, segmentation, and classification steps were kept the same in all setups. Moreover, to ensure generalisation of our findings, experiments were carried out on two benchmark pain datasets: the BVDB and the PainMonit Database (PMDB), a newly introduced dataset gathering physiological data of 52 participants subjected to heat-induced pain and annotated with both subjective and objective pain labels. To the best of our knowledge, we are the first to also record the subjective feedback of participants allowing us to create further findings in the research field of automated pain recognition.

The remainder of the work is organised as follows. Section 2 provides an overview of past approaches addressing the problem of automated pain recognition. In Section 3 the used material and methods are presented. The two pain databases with the BVDB and newly recorded PMDB dataset, pre-processing, segmentation, feature extraction, classification, and evaluation procedures and details on implementation are described in detail. The experimental results are summarised in Section 4. A discussion of the work is expressed in Section 5. Finally, Section 6 concludes the paper and displays some insights into future work.

## 2. State of the Art in Pain Recognition

Since high-quality annotated data is important to successfully perform supervised learning, many researchers have built and proposed their own benchmark datasets in the past pain literature. Such datasets mainly differ in terms of sensor modalities used to record the data and in terms of pain induction methods. Two main categories of modalities can be found: *behavioural* modalities record the manner and reactions of the participants, for example, with the use of video cameras. On the other hand, *physiological* sensors aim to capture biological responses of the body to pain. These reactions mainly arise from the autonomic nervous system and cannot be controlled consciously. The past literature has shown that: (1) pain influences the conductance of the skin, the heart rate, and specific muscles related to the inner tension measured as Electrodermal Activity (EDA), Electrocardiogram (ECG), and Electromyogram (EMG) respectively. (2) physiological signals seem to consistently perform better than behavioural modalities in automated pain recognition [23]. For those reasons, the work presented in this paper focuses on physiological sensors only. Various pain induction approaches have been used with physiological modalities in the past. Examples include stimuli-based methods, for instance using heat, electrical or cold pressure pain, or pain through exercises, for example, for people with shoulder trouble.

The most famous and widely used of all pain benchmark datasets is the BioVid Heat Pain Database (BVDB), published by Walter et al. [24] in 2013, which was the first dataset dedicated to the classification of heat-induced pain using physiological sensors and RGBD video recordings. In the following years, researchers evaluated various feature extraction methods on it. Most past work investigating feature extraction can be categorised into two classes: (1) *Feature engineering* strategies focus on manually extracting features of the data based on expert knowledge, thus referring to Hand-Crafted Features (HCF). Because of good performances and simplicity, these approaches were the standard techniques for a long time. (2) *Feature learning* involves algorithms automatically learning features from the given input data. Nowadays, the most popular feature learning approaches are based on deep learning that aims at solving machine learning tasks with Neural Network (NNs). NNs are mathematical models composed of simple non-linear computational units called artificial neurons, organised in a layer-wise structure, that can be used for various tasks including classification and regression. Past research (in particular in image classification) has shown that training a NN for a classification problem makes its neurons learn specific features (increasingly abstract the deeper the layer is) [25]. To achieve learning of meaningful features, models are trained in an end-to-end manner and have recently achieved outstanding performance in various classification tasks.

The first evaluation of the performance of a Support Vector Machine (SVM) exclusively based on HCF derived from physiological modalities was carried out by Walter et al. [26]. 135 features in 6 mathematical categories (amplitude, frequency, stationarity, entropy, linearity, and variability) were derived from the pre-processed signals. Moreover, a forward selection and backward elimination performed on the feature set was implemented. While the forward selection outperformed the backward one, a best accuracy of 77.05% for the task *no pain* vs. *high pain* in a Leave-one-subject-out (LOSO) setup could be obtained. One year later, Gruss et al. [27] strove to improve those results by extending the feature set with *similarity* features that try to estimate the resemblance between the mean baseline signal and sensor data under pain of the subject and helped to increase classification performance. Unfortunately, no LOSO results were published which makes a comparison to other work difficult. Kächele et al. [28] expanded the processing pipeline by a post-processing step where features were standardised per person and signal. Furthermore, a personalisation step by training only on the most similar person to the test set was implemented. A best accuracy of 85.7%. in a LOSO was reported. While it could be shown that the procedure improves the performance greatly, it relies on an *offline* setup. Real-time classification of unseen data is not possible as the complete database must be seen and processed first. Individual sensor analysis concluded that EDA yields the best performance and thus contains the most information of pain analysis. Amirian et al. [29] were the first to try feature learning using Radial Basis Function neural networks. Using EDA decomposition, a best accuracy of 85% for a LOSO was retrieved. In addition, a regression task was also tested and yielded a 6.7175 Root Mean Square Error (RMSE). Recent papers have aimed at finding more elaborate feature learning approaches purely based on deep learning. In [30] the authors evaluated several Convolutional Neural Network (CNN) architectures using different fusion approaches for the first time. It could be shown that previous approaches relying on HCF [28,31,32] can be outperformed and that the EDA signal was contributing the most. A CNN model trained on the EDA signal exclusively and a fusion approach consisting of a trained average weighting of three CNNs (one for each sensors modality) yielded 84.57% and 84.40% in a LOSO, respectively. Moreover, Thiam et al. [33] tested feature learning using Deep Denoising Convolutional Autoencoder (DDCAEs) and evaluated three distinct architectures using different embedding approaches. A single representation for each channel, a single and shared representation for all input channels, and a single representation using a gating layer to create a weighted output across channels were used as layers to embed features in a single feature vector. The DDCAEs were trained simultaneously with an additional NN classifying data samples based on the output of the encoder part. Significantly superior results were achieved with the gated latent space model and a best accuracy of 83.99% in a LOSO could be yielded.

## 3. Materials and Methods

The following section introduces the evaluation framework used to compare various feature extraction approaches in detail. To create a fair comparison of feature extraction methods we defined an experimental setup, that is used on several datasets with different labelling strategies. The data acquisition, pre-processing, segmentation, and classification steps of the pattern recognition chain were fixed (for each dataset) to capture the impact of different feature extraction approaches on the evaluation result.

### 3.1. Data Acquisition

During data acquisition, a dataset is gathered with a fixed sensor setup. Such data is indispensable for the later processing and training of the classification system. Moreover, ground truth is acquired to provide annotations - also referred to as labels - to the data. Labels provide information about which class each data example can be associated with. Section 3.1.1 and Section 3.1.2 respectively introduce the BVDB and PMDB datasets used in our experiments.

#### 3.1.1. BioVid Heat Pain Database (BVDB)

The BioVid Heat Pain Database (BVDB), published by Walter et al. [24] in 2013, represents the first *machine learning* dataset dedicated to the classification of heat-induced pain using physiological sensors and video recordings. 90 subjects with 30 people in each of the age groups 18–35, 36–50, and 51–65 participated in the experiment. Care was also taken to ensure that each age group consisted of the same number of males and females. Pain was induced via a thermode (Medoc, Ramat Yishai, Israel) at the right arm. The complete experiment consisted of 6 individual steps. During a *calibration phase*, pain ($T_{P}$) and pain tolerance ($T_{T}$) thresholds were found initially by slowly increasing the temperature beginning from 32 ${}^{\circ }$C. $T_{P}$ and $T_{T}$ describe the temperature when changes from heat to painful and bearable to unbearable pain happen for each subject, respectively. The interval $\left[T_{P},T_{T}\right]$ was then divided into four sub-intervals $\left[T_{i},T_{i+1}\right]$ where $T_{i}=T_{P}+\left(\left(i-1\right)\times R\right)$ for i∈{1,2,3,4} with $R=(T_{T}-T_{P})/4$. All temperatures $T_{i}$ defined this way were used during the stimulation phase to induce pain of variable intensity. *Pain stimulation* was performed by applying the 4 different temperature stimuli over the course of 25 min. Each temperature was applied 20 times for 4 s with an 8–12 s randomised pause in-between. Afterwards, a second *Pain stimulation (II)* phase with a different sensor setup was performed, resulting in the two available parts A and B of the BVDB. While Part A contains video, Skin Conductance Level (SCL), ECG, and EMG (Trapezius) information, Part B added EMG for the Corrugator and Zygomaticus muscles and dropped video sources as wires occluded parts of the faces. Since Part A represents the most commonly cited one, we decided to evaluate our models on this fraction of the dataset for better comparability. Thus, when speaking about BVDB, we refer to part A from now on.

To acquire a dataset with rich information, a camera setup and various physiological sensors were used to record pain responses. A setup of three *AVT Pike F145C* cameras and one Kinect Sensor was used. One camera with the Kinect sensor on top was placed directly in front of the participants to record RGB and depth information, respectively. To ensure that subjects were able to move their heads freely and while properly capturing facial expressions at all times, two additional cameras were placed at a $45^{\circ }$ angle to the left and right of the subject. Moreover, various physiological sensors were registered with the help of a *Nexus-32 amplifier*. The different modalities used for pain recognition are listed below:

- Electrodermal Activity (EDA): Two electrodes were placed on the index and ring finger to measure the skin conductance level also referred to as Galvanic Skin Response or SCL.
- Electrocardiogram (ECG): The participants’ heart rate activity was recorded using two electrodes, one on the upper right and one on the lower left of the body.
- Electromyogram (EMG): Muscle activity of 3 different sites were captured using two channel surface Electromyogram (sEMG). Electrodes were placed on two muscles in the face (Corrugator, Zygomaticus) and one on the shoulders (Trapezius).

Moreover, the data of three subjects were removed due to technical issues during the recording [31], resulting in a dataset of a total of 87 subjects. All sensor channels were resampled to a common sampling rate of 512 Hz. The dataset consists of already segmented windows of length of 5.5 s with a 3 s delay after the stimulus onset. Thus, a training sample of one sensor channel forms a vector with a length of samplingrate(512)×seconds(5.5)=2816. Additionally, each window of data was associated with a pain label between 1 (low pain) and 4 (high pain) depending on which stimuli $T_{i}$ (for i∈{1,4}) was applied during its acquisition. A baseline temperature $T_{0}$ set to 32 ${}^{\circ }$C was also used to obtain data related to no pain, leading to a total of five pain levels. By applying each temperature 20 times, there are 100 (stimuli (5) × repetitions (20)) data samples per subject.

#### 3.1.2. PainMonit Database (PMDB)

**Figure 2:**
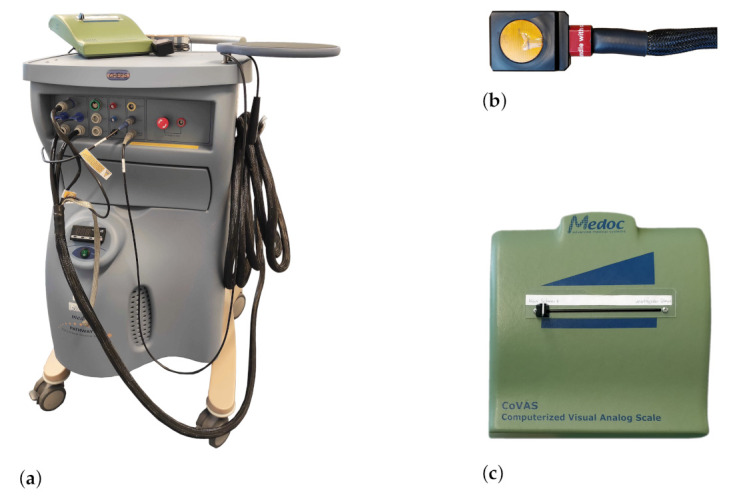
The Medoc devices used during data acquisition. (**a**) Medoc Pathway system. (**b**) Cheps thermode.(**c**) Computerised Visual Analogue Scale (CoVAS) slider.

The PainMonit Database (PMDB) was acquired at the Institute of Medical Informatics, University of Lübeck, Germany, following the findings of a preliminary study investigating heat-induced pain in a small dataset containing 10 subjects in Gouverneur et al. [34]. A Pathway CHEPS (Contact Heat-Evoked Potential Stimulator thermode, Medoc, Ramat Yishay, Israel) with a 27 mm diameter contact surface was attached to the non-dominant forearm interior site (10 cm below the elbow) of participants to induce pain by thermal stimuli as it is one of the most commonly used stimuli to induce experimental pain. In total 55 subjects (21 male and 33 female with an average age of 27.35±6.88) participated in the study. Healthy people between the age of 18 and 65 were recruited. In contrast, chronic pain disorders, acute pain, skin diseases that could be a contraindication to the thermode, pregnancy, neurological, psychiatric or psychological diseases, and regular use of medications (except contraceptives) were defined as exclusion criteria. The main difference between PMDB and existing benchmark in the literature is the presence of subjective pain annotations in addition to the objective temperature-based ones. Subjective feedback was obtained using a Computerised Visual Analogue Scale (CoVAS) slider (Computerized Visual Analogue Scale, Medoc, Ramat Yishay, Israel), a simple slider whose position is digitalised and returns ratings between 0 and 100. Like an ordinary VAS, the far left location represents *no pain* while the far right is associated with the *worst pain imaginable*. The pain induction machine, the thermode and slider can be seen in Figure 2a–c, respectively.

**Figure 3:**
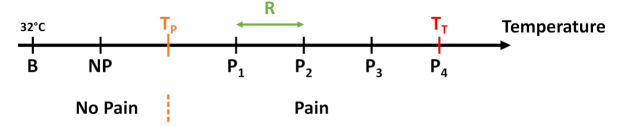
The 5 temperature intervals defined by the temperature range *R* in dependency of $T_{P}$ and $T_{T}$. While the first temperature resembles a non-painful stimulus, the last 4 are meant to evoke pain.

The new data acquisition protocol includes a *calibration* and *induction phase*. The calibration is based on recording the parameters pain threshold ($T_{P}$, threshold when heat stimulus becomes painful) and pain tolerance threshold ($T_{T}$, threshold when pain becomes unbearable) individually for each subject. Following a staircase calibration method, increasing 10-s temperature stimuli with 5 s pause in-between were given to the subject. The protocol started with a temperature of 40 ${}^{\circ }$C and increased the stimulations by 1 ${}^{\circ }$C each time up to a maximum of 49 ${}^{\circ }$C. Participants were asked to continuously rate their pain perception utilising the CoVAS. Temperatures exceeding 0 and 90 in CoVAS rating for the first time were noted as $T_{P}$ and $T_{T}$, respectively. To ensure further robustness of the calibration, these parameters were recorded twice for each subject and averaged. Subsequently, the thresholds were further tested to check their validity. During a calibration check, $T_{P}$ and $T_{T}$ were once again applied and rated by participants. If the former threshold was perceived as vigorously painful (CoVAS above 10), it was adjusted by reducing it by 1 ${}^{\circ }$C. Equally, $T_{T}$ was raised by 1 ${}^{\circ }$C if its initial value did not retrieve CoVAS values above 90. Four painful temperature stimuli $P_{i}$ were then defined using the thresholds $T_{P}$ and $T_{T}$ with the following equation:

(1) $$P_{i}=T_{P}+\left(i\times R\right)$$

with i∈{1,2,3,4} and $R=(T_{T}-T_{P})/4$. Moreover, a non-painful temperature NP was defined by $NP=T_{P}-R$. Figure 3 illustrates baseline, non-painful and painful temperatures with the associated thresholds.

During the pain induction phase, eight 10-s stimuli were applied for each of the temperatures $P_{i}$ defined previously. Between randomised stimuli, the temperature returned to the baseline at 32 ${}^{\circ }$C, and a resting phase of random duration between 20 to 30 s was applied. Participants were asked to rate their pain continuously using the CoVAS. To avoid any possibility of harm and sensitisation or habituation effects the thermode was repositioned after half of the impulses.

During the pain induction phase, various physiological sensors were recorded by two different wearable devices. Data of both were transferred via Bluetooth to one machine in real-time. On the one side, the wristband Empatica E4 (E4) (Empatica E4, Empatica Inc., Boston, United States) was worn on the non-dominant arm to avoid movement artefacts and recorded Blood Volume Pulse (BVP) from which Heart Rate (HR) and Inter-Beats-Interval (IBI) are computed, EDA, Accelerometer (ACC), and skin temperature in 64, 4, and 32 Hz, respectively. On the other side, respiBAN Professional (RB) (respiBAN Professional, Plux, Lisbon, Portugal), a chest-worn device registering respiration and various physiological modalities with a sampling rate of 1000 Hz, was included. Two electrodes were placed at the medial phalanx of the index and middle finger of the non-dominant arm to capture EDA. Moreover, the activity of the heart was measured by monitoring ECG with a positive electrode at the upper left, a negative electrode at the upper right pectoral and a reference electrode placed at the right waist. In addition, an electrode placed on the skin above the *trapezius* muscle recorded its activity via Electromyography (sEMG). To further reduce noise and artefacts a reference electrode was placed above the 7th cervical vertebrae. Moreover, the same self-adhesive disposable electrodes (Kendall Covidien H124SG ⌀ 24 mm, Wolfram Droh GmbH, Mainz, Germany) were stuck on all sites. No additional gel or other substance was needed, as the Ag/AgCl sensor is embedded in an adhesive ad conductive hydrogel. While the application takes a little longer than simple dry electrodes with a Velcro strap, Ag/AgCl hydrogel electrodes stick safely and provide the most reliable EDA signals [35]. As washing hands decreases skin conductance because it removes sweat and other conductance increasing substances, participants were asked to wash their hands with simple soap immediately before the procedure to standardise the time since the last handwashing ([36], p. 657). To further restrict the noise level, EMG and ECG sites were cleansed with simple alcohol pads [37].

In addition to the physiological sensors, behaviour responses were also recorded. An *HD Webcam Pro C920* (C920, Logitech, Lausanne, Switzerland) placed in front of the subjects, captured RBG-video information incorporating facial movements. Moreover, depth information was gathered using an Intel^®^ RealSense™ D435 (D435, Intel Corporation, Santa Clara, CA, USA) camera. To ensure consistent and sufficient lighting, two light boxes were setup in a $45^{\circ }$ to the left and right in front of the participant and shutters of the windows were closed. While aiming for a comparable setup, the same video modalities like BVDB (RGB and depth information) were recorded, but differences in devices (e.g., D435 vs. Kinect) could introduce differences in the resulting datasets. Subjects were asked to sit comfortably, rest the non-dominant arm and use the other to rate their pain using the CoVAS. The study took roughly one hour for each subject. Because of technical issues during the recording or flawed conduction of the experimental setup, three subjects were removed, creating a final dataset of 52 subjects in total.

### 3.2. Pre-Processing & Segmentation

#### 3.2.1. BioVid Heat Pain Database

**Figure 4:**
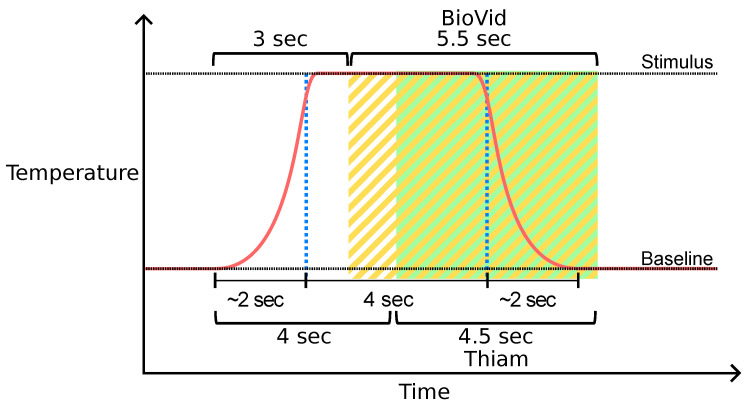
Window segmentation of the BioVid Heat Pain Database (BVDB) dataset. Window segments available in the public BVDB are highlighted in yellow. Thiam et al. [38] proposed another segmentation process highlighted in green that is used in [30,33] as well.

Since the BVDB is already segmented, no further segmentation step was implemented. Nevertheless, it is noteworthy that previous work proposed to realise different segmentation procedures. Thiam et al. [38] proposed to extract windows of 4.5 s with a shift from the elicitations’ onset of 4 instead of 3 s. Thus, presented and previously published results on the BVDB may not be directly comparable. Both segmentation approaches (original segmentation of the BVDB and the one proposed by Thiam et al. [38]) are visualised in Figure 4.

Kächele et al. [28] showed that *z-score normalisation* can boost performance on the BVDB, but the approach is only available as an offline procedure as standardisation was done per subject. To benefit from these findings, but at the same time being able to keep the system dynamic, a normalisation per window was conducted. In detail, a *min-max normalisation* [39] was used to transform the data to the range [0,1] as the approach has the benefit to retain the original distribution in contrast to a z-score normalisation [40]. To further reduce computational cost the raw data have been resampled to a common frequency of 256 Hz resulting in data samples of size time (*T*) × sensor (*S*) with T=1408 and S=3 including EDA, ECG and sEMG. Since previous work showed the importance of the EDA signal outperforming other single sensor modalities for the automated recognition of pain, this paper focuses on the classification of pain based solely on said channel. Thus, the data frames were further selected to just include the EDA signal, resulting in frames of shape 1408×1. No additional pre-processing step was performed to keep the computational pipeline minimal.

#### 3.2.2. PainMonit Database

In a first step, the CoVAS and temperature labels recorded by the *Medoc* software and collected sensor data of the PMDB were synchronised, resampled to a frequency of 256 Hz (similarly to the BVDB) and linearly interpolated to ensure that channels have a common recurrence. For the segmentation of the acquired data records, the whole duration of the pain stimuli, i.e., 10 s, was used to define the segment length. Like the BVDB the applied temperatures NP/$P_{1-4}$ were used as objective class labels for their associated data samples. Furthermore, 10-s windows preceding each stimulus were extracted and labelled with the non-painful class *B* because temperature remained at baseline during these segments.

**Figure 5:**
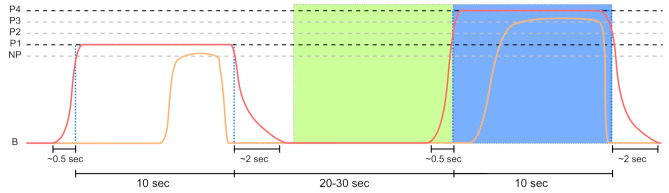
Sensor data segmentation of 10 s non-painful (green area) and painful (blue area) windows for the PainMonit Database (PMDB). The windows are centred around the on and offsets of the temperature (red curve) stimulus. Moreover, the CoVAS (orange curve) values are used to create a pain label that incorporates the subjective sensation of the subjects.

To assign a subjective pain label to each window, CoVAS values were processed in several steps. First, the sum of the CoVAS ratings for each segment was computed. The CoVAS sum associated with each segment was then scaled by dividing it by the maximum CoVAS sum obtained among all segments associated with the current subject. Next, discrete ranges were used to create the class labels where $C_{0}$ corresponds to the value 0 and $C_{1}$, $C_{2}$, $C_{3}$, and $C_{4}$ correspond to ]0,0.25], ]0.25,0.5], ]0.5,0.75], and ]0.75,1], respectively resulting in an additional dataset. The aforementioned steps of scaling and converting values into ranges were both performed in a subject-dependent way, i.e., per participant. Windows without any CoVAS response were associated with the class label $C_{0}$. The segmentation process is visualised in Figure 5. The obtained data frames were of size T×S with T=2560 and S=9 having 5 sensor channels for the E4 (BVP, EDA, skin temperature, IBI and HR) and 4 for the RB (respiration, EDA, ECG and sEMG). Again, data frames were filtered to only use the EDA information resulting in data frames of size 2560×1, 2560×1, and 2560×2 for the EDA derived from the RB, E4 and both, respectively.

Moreover, the same normalisation step done on the BVDB was performed on the segments of the PMDB as well.

### 3.3. Feature Extraction

The following Section 3.3.1 describes the calculation of HCF used in this study for pain recognition in detail. Section 3.3.2 summarises the most common supervised feature learning and Section 3.3.3 presents unsupervised feature learning approaches. As shown in previous work, the EDA signal provides the best classification results and is focused on in this work.

#### 3.3.1. Hand-Crafted Features

Traditional approaches for HCF extraction based on EDA data focus on the decomposition into its underlying parts, the tonic and phasic elements of the signal. Several varying methods have been introduced in the past to accomplish this process. In addition, more recent studies aimed to extract characteristics emphasising spectral analysis as well [35]. The following paragraph summarises the applied feature extraction approaches.

**Figure 6:**
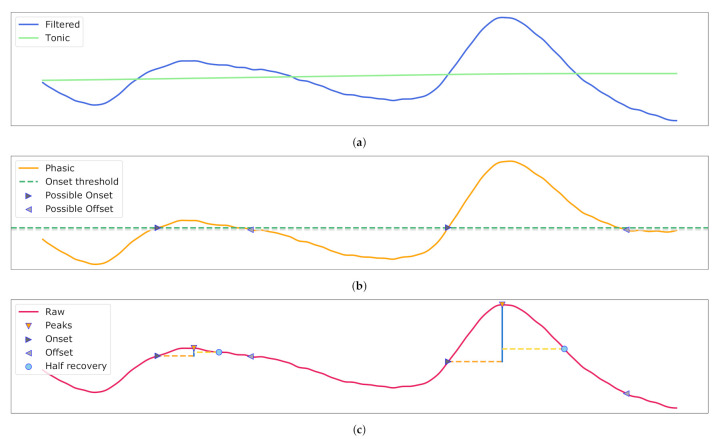
Electrodermal Activity (EDA) decomposition into the phasic and tonic signal with galvanic skin response detection. (**a**) The filtered input and retrieved tonic signal. (**b**) The phasic component with possible on- and offset for the peaks. (**c**) The raw signal with Skin Conductance Response (SCR) and their associated peaks, half recovery, on- and offsets.

Initially, the EDA signal was split into its *phasic* and *tonic* components to investigate the rapid changing spikes, also called Skin Conductance Response (SCR) and slowly adapting SCL, respectively. While SCL describes the current degree of conductance, which changes gradually, SCRs are frequently occurring spike-shaped peaks in the EDA signal. They result from autonomic nervous system arousal in response to a stimulus and thus are also referred to as event-related skin conductance responses. To decompose the sensor data a simple approach of a forward-backward digital filter using cascaded second-order sections was used. A second order butterworth with a cutoff frequency of 0.05 was chosen. Applying the filter as low-pass and high-pass filter yields the tonic and phasic components [41]. Moreover, SCRs are found in the phasic part by determining a peak when an onset threshold of 0.01 and peak amplification threshold of 0.05 is exceeded [42,43]. Peak, half recovery, on- and offsets of each SCR are identified and example data are visualised in Figure 6.

Moreover, 38 distinctive features deriving from literature [44] were extracted from the window segments. An overview of the features can be found in Table 1 (later on referred to as “HCF” approach).

Furthermore, recently successful methods to extract hand-crafted features for automated pain recognition proposed in literature have been realised. The more sophisticated methods named derivative of phasic component of EDA (dPhEDA) based on a convex EDA optimisation method (cvxEDA) [45] and spectral features time-varying index of sympathetic activity (TVSymp) and its modified version (modified spectral features time-varying index of sympathetic activity (MTVSymp)) [46,47] have been implemented as well and were compared. Moreover, a feature fusion approach including all hand-crafted features coming from the different methods (HCF, dPhEDA, TVSymp and MTVSymp) was evaluated and is referred to as “HCF combined”.

#### 3.3.2. Supervised Feature Learning—Neural Networks

Deep learning approaches aim to automatically learn features from given input data. During training, the raw data is fed to NNs so they can learn a mapping towards the class output in an end-to-end manner. While these models have been shown to achieve state-of-the-art performances for various tasks (for example, image recognition) their training can be challenging as training is computationally expensive and finding optimal architectures is not trivial. While relying on countless simple calculations it is challenging to derive a human-understandable explanation of the models’ decision outputs, thus also referring to *black box* models.

**Figure 7:**
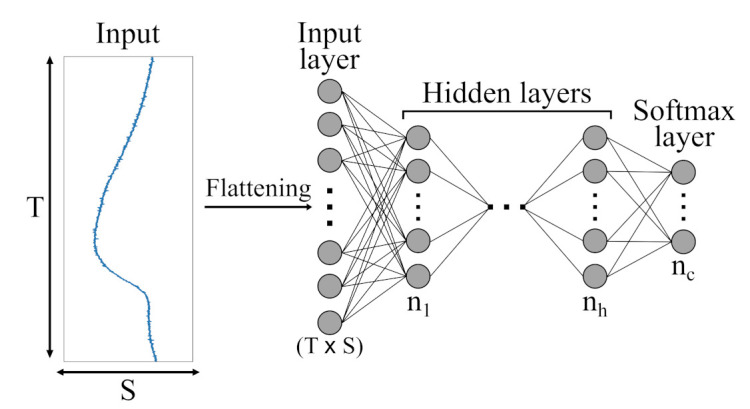
Schematic illustration of an Multi-Layer Perceptron (MLP) architecture with *h* hidden layers and *c* output classes presented by a softmax layer. Initially, the different sensor channels are flattened into a (T×S)-dimensional vector and then fed to the various hidden layers.

Different architectures, like Multi-Layer Perceptron (MLPs) [48], CNNs [49] and Recurrent Neural Network (RNNs) [50], have been adopted for various tasks such as natural language processing, classification, segmentation, image reconstruction, and time-series prediction in the past. MLPs represent the simplest type of NN. To handle 2-dimensional sensor data with its time (*T*) and sensor (*S*) axis, input data are flattened and presented to a dense layer that awaits 1-dimensional input. A schematic illustration of an MLP architecture can be found in Figure 7.

**Figure 8:**
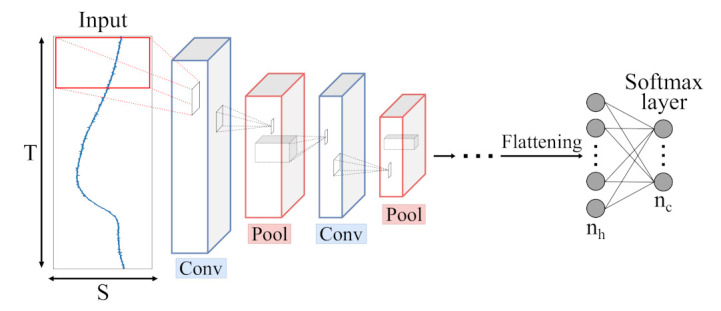
Schematic illustration of a Convolutional Neural Network (CNN) architecture with *c* output classes presented by a softmax layer. Input data is processed by Convolutional (blue) and Pooling (red) layers, extracting meaningful features which are fed to a combination of dense layers, similar to single MLP layers.

In contrast, CNNs, first introduced for image data, normally handle 3-dimensional data (width × height × channels). To process sensor data, the raw data are fed to CNNs as 3-dimensional inputs (T×S×1) in our study. For classification purposes, MLPs, consisting of several dense layers, are often appended to the convolutional network. An illustration of a CNN can be seen in Figure 8.

Furthermore, so-called Convolutional LSTM networks were introduced [51], utilising the benefits of Long Short-Term Memory (LSTM) and CNN layers at the same time by establishing a novel type of layer. Classic LSTM layers have been extended for this purpose. Instead of using internal matrix multiplications of MLPs, ConvLSTM layers replace them with convolution operations of CNNs architectures. These hybrid layers seem to capture spatiotemporal correlations better than classic LSTM layers whose dense build has too many redundant connections. To process these spatiotemporal relations, ConvLSTM layers (like simple LSTM layers) are fed with data of several time segments describing one sample. Afterwards, the time sequential fragments of the initial sensor data with equal size are interpreted one after another. Thus, the input data for ConvLSTM layers have an additional axis compared to simple CNN layers and are 4-dimensional (number of segments, time length, number of sensor channels, number of channels). Preliminary studies tested various values for the parameter “number of segments” and determined 4 to be the one returning the best performances. Therefore, the sensor information was split into 4 parts resulting in data frames with shape (N×T/N×S×1) with *N* being the number of segments or more specifically (4×352×1×1) and (4×640×1×1) for the BVDB and PMDB, respectively.

#### 3.3.3. Unsupervised Feature Learning—Convolutional Autoencoders

**Figure 9:**
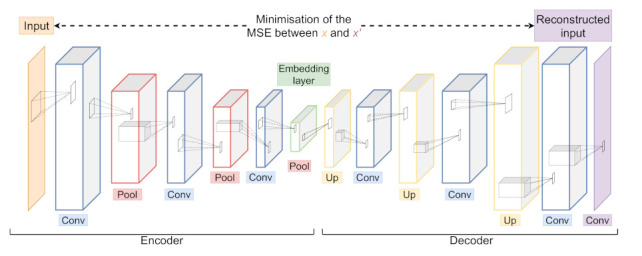
An example of a Convolutional Autoencoder (CAE) architecture with its Encoder and Decoder part consisting of Convolutional (blue), Pooling (red) and Upsampling (yellow) layers. During training, the network is optimised to minimise the difference between input *x* and output $x^{\prime }$. The embedding layer (green) yields a low dimensional representation of the input *x*.

Besides the presented neural networks for classification tasks, deep learning for unsupervised feature extraction has been investigated in the past as well. One of the most popular unsupervised feature learning approaches is based on Autoencoder (AEs) [52]. AEs are intended to learn a low-dimensional representation, also referred to as *encoding*, in an unsupervised way. The dimensionality reduction is enforced by its hourglass shaped architecture, comprising an *embedding* and *decoder* with a bottleneck in-between. While the encoder maps the input sample to a lower dimensional feature space, the decoder tries to reconstruct the original sample by upsampling the embedding. During successful training of an autoencoder, the output is close to being identical to the input, as the decoder can completely reconstruct the input from the given low-dimensional representation. To evaluate the differences between input and output, often the Mean Squared Error (MSE) is used as reconstruction loss. In addition to AE, Convolutional Autoencoder (CAEs) follow the same principle leveraging convolutional layers by reducing dimensionality with *pooling* layers and increasing dimensionality with *upsampling* layers. Upsampling layers can be seen as a reverse operation to pooling layers as they scale up the given input by repeating it. An illustration of a CAE can be seen in Figure 9.

### 3.4. Classifier

To ensure a fair comparison of the different aforementioned feature extraction approaches, an Random Forest (RF) [53] was chosen as unique classifier in every setup. Also, deep learning models, which usually combine the task of feature extraction and classification, are just used to extract features to then follow the same classification step as other approaches. To utilise deep learning models as feature extraction approach, supervised NN architectures were first trained as classifiers. Here, networks were fed with 3-dimensional data (T×S×1), in most of our studies focusing on a single EDA channel being (T×1×1). Afterwards, the classification layer (softmax) was removed to transform the model into a feature extractor with the initial penultimate layer outputting characteristics. The truncated model was then used to obtain feature vectors from the examples of the dataset to train the RF classifier. Similarly, the CAE was trained in an unsupervised way on the training set first. Afterwards, the encoder part was used as a feature extractor by transposing the dataset to the feature set which is used to train and test the RF again.

Although multi-class classification has been investigated in the pain literature, best performance results could be reported for binary classification tasks, where each class represents a specific pain level. The best performances could be obtained for the classification of very dissimilar pain levels, for example, *no pain* vs. *high pain*. Thus, classifiers are exclusively trained to distinguish the non-painful class against the painful classes of each dataset in our study. For BVDB we report $T_{0}$ vs. $T_{i}$ for i∈{1,2,3,4}, for PMDB *B* vs. NP/$P_{i}$ for i∈{1,2,3,4} and $C_{0}$ vs. $C_{i}$ for i∈{1,2,3,4}.

### 3.5. Evaluation

The evaluation methods were designed to match previous work and thus simplify comparison. Models were assessed in a Leave-one-subject-out (LOSO) Cross Validation (CV) scheme, where the data of each subject is used as a testing set once, while the rest of the dataset forms the training set. Overall performance is obtained by averaging the classification performances obtained for each tested subject. The protocol ensures that all models are tested on unseen subjects (subject-independent), providing a realistic estimation of the classifier used in real world applications.

The performance of such an experiment is estimated by the amount of correctly predicted positive labels (*tp*), amount of mistakenly predicted positive labels (*fp*), amount of correctly predicted negative labels (*tn*) and amount of mistakenly predicted negative labels (*fp*). To report the classification performances of the different tested feature sets, we used the accuracy, given as:

(2) $$Accuracy=\frac{tp+tn}{tp+tn+fp+fn}$$

Another performance evaluation can be given by the $F_{1}\;score$ (Equation (5)). The $F_{1}\;score$ is defined by the harmonic mean between precision (Equation (3)) and recall (Equation (4)) and can fairly evaluate a setting with a class imbalance.

(3) $$Precision=\frac{tp}{tp+fp}$$

(4) $$Recall=\frac{tp}{tp+fn}$$

(5) $$F_{1}\;score=\frac{2\times Precision\times Recall}{Precision+Recall}$$

To be more precise, the $macro\;F_{1}\;score$ which consists of the average of all class $F_{1}\;score$ is reported in our experiments:

(6) $$macro\;F_{1}\;score=\frac{1}{n}\sum_{i=1}^{c}F_{1}\;score_{i}$$

where *c* is the number of classes and $F_{1}\;score_{i}$ constitutes the $F_{1}\;score$ for the *i*th class. Thus, from now on when presenting $F_{1}\;scores$ we display the $macro\;F_{1}\;score$.

### 3.6. Implementation Details

Instead of reporting the outcome of one single LOSO, we decided to indicate the average results obtained after performing LOSO five times. This was motivated by the fact that outcomes of the deep learning methods showed that they fluctuate with different iterations due to randomness in weight initialisation. Moreover, the bootstrapping process in RFs further introduces a small variance in the results.

All algorithms and models were implemented using Python. For the RF and Deep learning architectures, the *sklearn* and *Keras* with *Tensorflow 2.2.0* backend libraries were used, respectively. In our setup, the RF implementation of the package *sklearn.ensemble* with 100 diverse trees was realised. As optimiser for NN architectures, the Adaptive Moment estimation (ADAM) [54] with an initial learning rate of $10^{-4}$ was chosen and models were trained with 50 epochs using a batch size of 8. MSE was used as a loss function for the CAEs experiments, *categorical cross-entropy* for the rest. As automated hyper-parameter optimisation remains to be an obstacle [55], all architectures and their associated parameters have been found and optimised in a trial-and-error manner. The average accuracy obtained in a 5 × LOSO setup for the tasks no pain (*B*) vs. high pain ($P_{4}$) of the PMDB was used as evaluation metric to select the best performing architecture on all subjects on average for each deep feature learning approach. For example, for the CNN approach different numbers (1, 2, 3) of blocks (one block consisting of a Convolutional, Max pooling and Dropout layer) and various numbers of filters (10, 16, 32, 64) were tested, yielding best results with 2 blocks and 16 filters. Moreover, the CNN architecture for EDA signals proposed by Thiam et al. [30] was implemented and compared to our approaches using the presented setup. While Thiam et al.’s model uses a deeper architecture than ours, it was not able to retrieve better results and thus was not evaluated in later experiments. A report of the results it could achieve on the BVDB and PMDB can be found in the appendix (Appendix A). Similarly, MLPs with different numbers of blocks (1, 2, 3) were tested, one block consisting of a Dropout and Dense layer with different numbers of neurons (50, 100, 250, 500) having little impact on the results with the best configuration at one block with 250 neurons. Our CAE architecture is inspired by the one presented in [33] and slightly optimised by using Rectified Linear Unit (ReLU) as activation, increasing the pooling size and adopting the number of filters. Description of the different architectures can be found in Table 2, Table 3, Table 4 and Table 5, describing the layer type and their properties, e.g., a ReLU activation. The layers presenting features to the RF in the chosen classification pipeline are highlighted in grey.

## 4. Results

The following section presents the results of the various experiments, first for the BVDB, then for the PMDB. Models were trained in a binary classification task opposing a specific pain level with a non-painful baseline. Table 6 summarises the 5× LOSO average evaluation metrics for RFs trained on different feature extraction approaches just using the EDA signal. While the MLP approach yields the best results (84.01%±14.01 for $T_{0}$ vs. $T_{4}$) for all tasks, the gap between the performances of the different methods remains small (especially for deep learning and the best hand-crafted feature approaches). For $T_{0}$ vs. $T_{4}$ MLP and “HCF combined” return the best outcomes for deep learning and hand-crafted feature methods respectively with a difference of 0.64% in accuracy. MLP and CNN are the best and worst performing deep learning models with a difference of 1.08% in accuracy.

Despite less advanced pre-processing involving only a per-window data normalisation, our features achieve similar performances to those reported in the literature. A comparison of the best LOSO performances obtained by our MLP approach to earlier work is given in Table 7. To the best of our knowledge, Thiam et al. [30] still present the best performing results for deep learning methods on the BVDB. The minor performance gap between their and our results could be argued due to their advanced data pre-processing, including several filters, segmentation, and data augmentation which were not in the scope of this paper. In addition, using an end-to-end trained NN with a softmax layer for classification could have advantages over the RF classifier.

In contrast to the BVDB, the PMDB includes two sources for the EDA, having one site collected at the wrist by the E4 and one at the medial phalanx of the index and middle finger by the RB. Thus, all EDA sensor combinations with an MLP as a classifier were evaluated in a first test by feeding the network with data samples of shape (T×S×1). Table 8 shows a comparison of the EDA sensor coming from the E4 (EDA_E4) and RB (EDA_RB) and an early fusion of both. For the fusion approach, the information of both devices was supplied to the classification approach as one data frame (T×2×1) simply by concatenating the data. The outcomes suggest that EDA_RB outperforms EDA_E4 by being significantly better in all tasks but *B* vs. NP. Moreover, the fusion of both yields only better accuracy results for *B* vs. NP/$P_{1}$/$P_{2}$ (only significantly better for NP/$P_{2}$), with EDA_RB still performing significantly better when used alone for *B* vs. $T_{3}$/$T_{4}$. Thus, EDA_RB outperforms EDA_E4 and the fusion of both for most tasks making the expense of the merging inefficient. Further experiments therefore report only results using EDA_RB.

For easier comparison with the results obtained on the BVDB, the results associated with the objective temperature labels on the PMDB are provided first. Table 9 summarises the results. While the BVDB does not contain data for the *B* vs. NP problem, the tasks *B* vs. $P_{1-4}$ are somehow comparable to $T_{0}$ vs. $T_{1-4}$. Again, the margin between the various approaches stays minimal between HCF and deep learning approaches. The mean accuracy across methods for *B* vs. NP/$P_{1-4}$ are around ≈49%,57%,63%,72% and 86% respectively. For task *B* vs. $P_{4}$, the CNN yields the best accuracy of 87.41%±11.99 for a 5× LOSO average performance. Outcomes of task *B* vs. NP remain close to a random guess (50% in a two-class problem) for all extraction methods.

Lastly, Table 10 provides an overview of the results obtained after using the subjective CoVAS labels of the PMDB dataset. An increase in accuracy and $F_{1}\;score$ can be seen for all tasks. A best accuracy of 93.78% and an $F_{1}\;score$ of 87.60% can be reported for $C_{0}$ vs. $C_{4}$ using the HCF approach.

## 5. Discussion

The following section offers a detailed discussion concerning the presented results. Especially, the tasks no pain vs. high pain ($T_{0}$ vs. $T_{4}$, *B* vs. $P_{4}$, $C_{0}$ vs. $C_{4}$), retrieving the best performances in the past and our work, are analysed. The aforementioned results lead to the following conclusions. In contrast to what previously published work suggest [30], well engineered HCF still yield relevant performances compared to deep learning approaches on both datasets. This is underlined by the fact that the difference in accuracy between “HCF combined” and the best approach is 0.64% for $T_{0}$ vs. $T_{4}$ while being 0.2% between HCF and the best deep learning approach for *B* vs. $P_{4}$. Although more sophisticated HCF approaches, like dPhEDA, TVSymp and MTVSymp show decent performance for several tasks, they perform slightly worse compared to the presented HCF vector. Nevertheless, they seem to be complementary and thus can improve results, for example, on the BVDB where “HCF combined” performs better than “HCF”. Possible improvements could be achieved by further adopting and generalising these features as dPhEDA, TVSymp and MTVSymp have been optimised and evaluated on datasets including a divergent pain induction, sensor setup and segmentation process compared to ours. Especially, the methods have been recently tested on larger time windows (25 and 55 s in [46]) compared to ours. Nevertheless, our accuracy results for no pain vs. high pain using the three approaches, i.e., ranging from 78.46% to 81.66% for biovid ($T_{0}$ vs. $T_{4}$) and ranging from 79.44% to 84.71% for painmonit (*B* vs. $P_{4}$), are somehow similar to previously published results on the thermal grill (TG) dataset. Here, using an RF in a LOSO evaluation the best accuracy of 81.5% for no pain vs. high pain was published in [46]. Moreover, no approach is significantly better than HCF for the task *B* vs. $P_{4}$. Thus, future work on automated pain recognition should focus on fusing feature engineering and learning methods to further boost classification achievements. In addition, the narrow gap between the performances of the different feature extraction methods indicates that the required information for automated pain classification relying on the EDA signal is simple to retrieve, and most reported techniques can find them. This is emphasised by the absence of approaches significantly outperforming others for all tasks (except dPhEDA, TVSymp and MTVSymp). For *B* vs. $P_{4}$ an STD of 0.41 and for $T_{0}$ vs. $T_{4}$ an STD of 0.48 across the 5× LOSO accuracy of the HCF and deep learning methods was measured. Moreover, this leads to the deduction that more complex deep learning architectures do not necessarily perform better than simpler ones, as our simple MLP yields the best results for BVDB and close to best for PMDB (0.91% difference in 5 × LOSO accuracy mean with the best performing approach) in comparison to architectures involving more complex layers.

Moreover, outcomes generated on the PMDB confirm results reported on the BVDB in the past and generate new insights. Slightly better but similar results can be obtained on PMDB for the different temperature tasks. The enhanced classification performance could be caused due to several factors. On the one hand, differences in the study protocols could explain the performance gap. In particular, the redundant control of temperature thresholds in the calibration phase makes the PMDB study protocol more robust. On the other hand, the stimulus duration (BVDB: 4; PMDB: 10 s) was increased and could possibly harvest more precise high pain data samples, as it has been shown that “heat pain is assessed more reliably in tonic stimuli compared to phasic” [57].

Due to a modified setup, the newly recorded PMDB has the capability to generate new insights in automated pain recognition. In contrast to other pain databases, wearable recording devices, a non-painful stimulus and a CoVAS slider were introduced. Firstly, the results for task *B* vs. NP show that it remains an obstacle to distinguish between a non-painful stimulus and no stimulus at all. Further, these findings suggest that the success of discriminating no pain from pain in our setup is related to the pain itself rather than the recognition of physiological responses to any applied stimulus. Thus, features retrieved from SCL are associated with the painful temperature and not just an event-related response. Secondly, the exclusive use of two wearables to capture physiological modalities and classification results showed that these mobile devices have the capability of recording the underlying information sufficiently and create the possibility to generate a mobile build. Thirdly, the promising results of CAE indicate that meaningful features can be extracted in a completely unsupervised way. Finally, the novel acquired CoVAS values enable fresh insights. While the average $F_{1}\;score$ performance using HCF is 86.67% for *B* vs. $P_{4}$, it improves to 87.60% for the task $C_{0}$ vs. $C_{4}$. The greater classification performance on labels incorporating the subjective feedback of participants suggests that crucial information is given there. As stimuli are perceived diversely due to sensitisation or habituation effects, COVAS ratings differ for the same applied temperatures. These variances and the corresponding physiological responses appear to be better constituted in the labels $C_{1-4}$ than in $P_{1-4}$, thus yielding better classification performance (significant improvement for the average $F_{1}\;score$ performance between $P_{4}$ and $C_{4}$ can be measured for RF and CAE).

**Figure 10:**
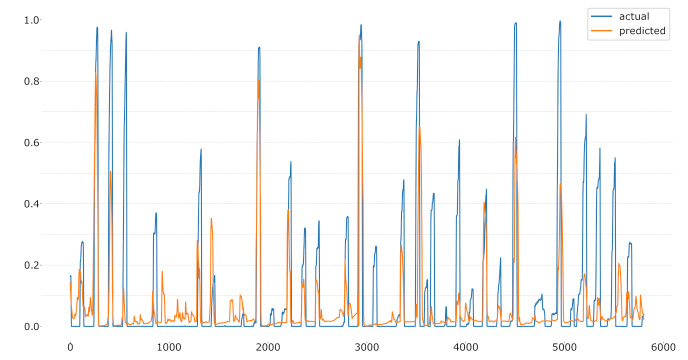
LOSO regression using the CoVAS values as label.

**Figure 11:**
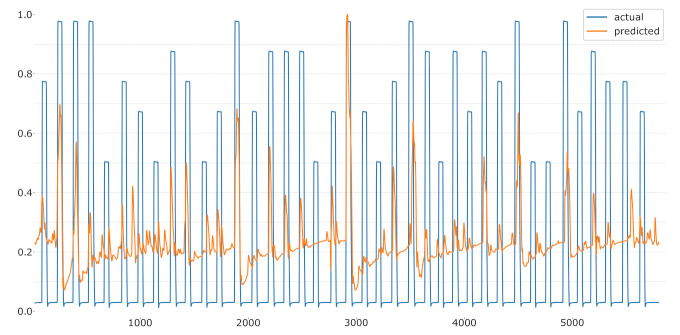
LOSO regression using the temperature values as label.

Moreover, the novel label facilitates new machine learning tasks. Having a continuous measurement, the classification problem can be transformed into a regression problem. Figure 10 and Figure 11 visualise possible regressions of the CoVAS and temperature data trained exclusively on the EDA signal in a LOSO setup for one subject. Again, the CoVAS values seem to yield better results achieving an MSE of 0.03 while the temperature label returns an MSE of 0.09. Detail investigations of the regression task could be addressed during future work.

## 6. Conclusions

In this paper, we introduced an evaluation framework allowing a fair comparison of feature extraction methods on physiological sensor data in the scope of automated pain recognition. By fixing the pre-processing, segmentation, and classification steps of the pattern recognition chain, the performances of some of the most popular feature extraction and feature learning approaches are compared. Experiments were carried out on the BVDB, the most popular benchmark dataset for pain recognition and the newly introduced PMDB dataset that is—to the best of our knowledge - the first to include subjective pain ratings. The results lead to the following findings: firstly, well engineered HCF still yield relevant performance compared to feature learning approaches relying on deep learning. Furthermore, more complex deep learning architectures do not necessarily perform better than simpler ones. In addition, the study using CoVAS labels showed that subjective feedback of participants can be used to train robust pain classification systems instead of objective measurement used in the past (like the applied stimuli temperature). Finally, wearable devices can capture the underlying information in physiological signals to distinguish high pain from no pain.

While providing new insights for the pain machine learning community, this paper exclusively focused on the EDA signal as previous work underlined it as being the most promising one. However, EDA responses are not specific to pain and could be triggered by other events as well, thus introducing a bias in classification predictions. Improvements by assessing novel sensor channels recorded by wearables of the PMDB and introducing dedicated fusion approaches will be addressed in a future iteration of this work. Moreover, Lopez et al. extensively researched the use of subject-clustering. A sophisticated HCF, deep learning, or fusion approach incorporating individual subject differences could possibly boost the performance of classification models. In addition, medical setups could benefit from transforming the task from classification to regression to provide detailed outcomes and information rather than presenting broad estimations of the class labels.

## Figures and Tables

**Table 1:** Features computed for the EDA signal.

Features
root mean square (RMS)
mean value of local maxima & minima
mean absolute value
mean of the absolute values (mav) of the first differences (mavfd)
mavfd on standardised signal
mav of the second differences (mavsd)
mavsd on standardised signal
variation of the first and second moment
indices of the minimum & maximum values; difference first and last value
mean & Standard Deviation (STD) for phasic, tonic, amplitudes,
rise times half recovery and recovery range of tonic; number of
Galvanic Skin Response (GSRs); sum of amplitudes; first amplitude; phasic max;
mean, STD and Variance (VAR) on normalised signal

**Table 2:** MLP architecture.

Layer Name	Neurons/Drop Rate	Activation
Flatten	-	-
Dropout	0.1	-
Dense	250	-
Dropout	0.1	-
Dense	100	-
Dense	2	Softmax

**Table 3:** CNN architecture with dropout rate set to 0.1.

Layer Name	No. Kernels (Units)	Kernel (Pool) Size	Stride	Activation
Convolutional	16	7	2	ReLU
Max Pooling	-	4	-	-
Dropout	-	-	-	-
Convolutional	16	7	2	ReLU
Max Pooling	-	4	-	-
Dropout	-	-	-	-
Flatten	-	-	-	-
Dense	100	-	-	-
Dense	2	-	-	Softmax

**Table 4:** The architecture that uses ConvLSTM blocks, later referred to as just ’LSTM’ with a drop rate set to 0.1. The ’return_sequences’ parameter for the ConvLSTM layers was set to True.

Layer Name	No. Kernels (Units)	Kernel (Pool) Size	Stride	Activation	Recurrent Activation
ConvLSTM2D	32	11	8	Tanh	Hard sigmoid
Dropout	-	-	-	-	-
Batch normalisation	-	-	-	-	-
Max Pooling (3D)	-	4	-	-	-
ConvLSTM2D	16	7	8	-	Hard sigmoid
Dropout	-	-	-	-	-
Batch normalisation	-	-	-	-	-
Max Pooling (3D)	-	4	-	-	-
ConvLSTM2D	8	3	2	-	Hard sigmoid
Dropout	-	-	-	-	-
Batch normalisation	-	-	-	-	-
Max Pooling (3D)	-	4	-	-	-
Flatten	-	-	-	-	-
Dropout	-	-	-	-	-
Dense	100	-	-	ReLU	-
Dense	2	-	-	Softmax	-

**Table 5:** CAE architecture with its encoder and decoder part.

Layer Name	No. Kernels (Units)	Kernel (Pool) Size
Convolutional	64	7
Max Pooling	-	4
Convolutional	32	11
Max Pooling	-	4
Convolutional	16	11
Max Pooling	-	4
Up Sampling	-	4
Convolutional	16	11
Up Sampling	-	4
Convolutional	32	11
Up Sampling	-	4
Convolutional	64	7
Convolutional	1	1

**Table 6:** 5× Leave-one-subject-out (LOSO) average performance using the EDA sensor of the BVDB for several feature extraction methods in combination with a Random Forest. Performance metrics are given as average accuracy (upper) and $F_{1}\;score$ (lower half) ± average standard deviation of each individual LOSO run. The best performing approach is highlighted in grey. Moreover, a paired student’s *t*-test with a significance level of 5% was performed to check the significance in differences between the accuracies obtained by each pair of approaches for each classification problem. ${}^{1,2,3,4,5,6,7,8,9}$ indicate a significant improvement compared to the HCF, dPhEDA, TVSymp, MTVSymp, “HCF combined”, MLP, CNN, LSTM, and CAE approaches, respectively.

Method		$T_{0}$ vs. $T_{1}$	$T_{0}$ vs. $T_{2}$	$T_{0}$ vs. $T_{3}$	$T_{0}$ vs. $T_{4}$
	HCF	$58.10\pm 12.81{\;}^{2,3,4,5,7,8}$	$63.27\pm 14.22{\;}^{2,3,4}$	$74.10\pm 13.53{\;}^{2,3,4,7}$	$82.73\pm 14.82{\;}^{2,3,4}$
	dPhEDA	$56.10\pm 10.02{\;}^{3,4}$	$61.28\pm 13.61{\;}^{3,4}$	$70.75\pm 13.62{\;}^{3,4}$	$81.66\pm 13.47{\;}^{3,4}$
	TVSymp	$53.83\pm 09.82{\;}^{4}$	59.15±12.49	$68.75\pm 13.80{\;}^{4}$	$79.51\pm 14.44{\;}^{4}$
	MTVSymp	52.86±10.43	$59.86\pm 12.45{\;}^{3}$	67.78±13.48	78.46±14.23
Acc	HCF combined	$57.49\pm 11.67{\;}^{2,3,4,8}$	$63.46\pm 14.30{\;}^{2,3,4,8}$	$74.18\pm 13.76{\;}^{2,3,4,7}$	$83.37\pm 14.19{\;}^{1,2,3,4}$
	MLP	$57.85\pm 12.05{\;}^{2,3,4,8}$	64.68 ± 14.06 ^1,2,3,4,5,7,8^	74.77 ± 13.79 ^2,3,4,5,7,8^	84.01 ± 14.01 ^1,2,3,4,5,7,8^
	CNN	$57.08\pm 11.70{\;}^{2,3,4}$	$64.05\pm 15.07{\;}^{1,2,3,4,8}$	$72.96\pm 13.96{\;}^{2,3,4}$	$82.93\pm 14.22{\;}^{2,3,4}$
	LSTM	$56.68\pm 11.38{\;}^{3,4}$	$62.51\pm 13.73{\;}^{2,3,4}$	$73.32\pm 13.98{\;}^{2,3,4}$	$83.48\pm 13.62{\;}^{1,2,3,4,7}$
	CAE	58.39 ± 12.31 ^2,3,4,7,8^	$64.35\pm 14.55{\;}^{1,2,3,4,8}$	$74.09\pm 14.11{\;}^{2,3,4,7,8}$	$83.70\pm 14.39{\;}^{1,2,3,4,7}$
	HCF	57.46±13.21	62.32±15.05	73.41±14.33	82.38±15.32
	dPhEDA	55.69±10.12	60.62±14.02	70.05±14.26	81.23±14.14
	TVSymp	53.41±09.88	58.47±12.98	67.96±14.57	79.12±14.93
	MTVSymp	52.47±10.49	59.19±12.91	66.95±14.19	78.08±14.65
$F_{1}$	HCF combined	56.97±11.83	62.52±15.10	73.39±14.65	82.97±14.84
	MLP	57.20±12.39	63.47 ± 15.10	73.96 ± 14.68	83.58 ± 14.78
	CNN	56.44±12.01	62.93±15.92	72.15±14.77	82.52±14.86
	LSTM	56.19±11.57	61.64±14.38	72.54±14.86	83.12±14.25
	CAE	57.77 ± 12.59 ^2,3,4,7,8^	63.33±15.41	73.30±14.96	83.34±14.98

**Table 7:** LOSO accuracy performance comparison to early work on the EDA signal of the BVDB. The best performing approach is highlighted in grey.

Method	$T_{0}$ vs. $T_{1}$	$T_{0}$ vs. $T_{2}$	$T_{0}$ vs. $T_{3}$	$T_{0}$ vs. $T_{4}$
Werner et al. [31]	55.40	60.20	65.90	73.80
Lopez-Martinez et al. [56]	56.44	59.40	66.00	74.21
Thiam et al. [30]	61.67 ± 12.54	66.93 ± 16.19	76.38 ± 14.70	84.57 ± 14.13
MLP (Ours)	59.08±12.67	65.09±13.71	75.14±13.49	84.22±13.86

**Table 8:** 5× LOSO average performance using different EDA sensor combinations of the PMDB with an MLP + Random Forest (RF) classifier. The best performing approach is highlighted in grey. Moreover, a paired student’s *t*-test with a significance level of 5% was performed to check the significance in differences between the accuracies obtained by each pair of approaches for each classification problem. ${}^{1,2,3}$ indicate a significant improvement compared to the EDA_RB, EDA_E4, and fusion approaches, respectively.

Method		*B* vs. NP	*B* vs. $P_{1}$	*B* vs. $P_{2}$	*B* vs. $P_{3}$	*B* vs. $P_{4}$
	EDA_RB	50.43±12.92	$57.55\pm 11.17{\;}^{2}$	$63.17\pm 12.63{\;}^{2}$	72.84 ± 14.27 ^2,3^	86.50 ± 12.76 ^2,3^
Acc	EDA_E4	$53.46\pm 12.92{\;}^{1}$	53.87±14.78	57.41±15.41	60.84±16.38	71.15±18.64
	Both	54.59 ± 12.97 ^1,2^	58.37 ± 13.39 ^2^	64.59 ± 13.60 ^1,2^	$71.47\pm 15.74{\;}^{2}$	$85.19\pm 12.75{\;}^{2}$
	EDA_RB	49.31±13.34	56.12 ± 11.49	61.86 ± 13.25	71.57 ± 15.37	85.82 ± 13.98
$F_{1}$	EDA_E4	49.79 ± 14.46	50.86±15.74	54.78±16.88	58.52±17.89	69.11±20.65
	Both	49.43±15.61	54.04±16.15	61.62±16.35	69.66±17.67	84.11±14.94

**Table 9:** 5× LOSO average performance using the EDA (RespiBan) sensor of the PMDB for several feature extraction methods in combination with a Random Forest. Performance metrics are given as average accuracy (upper) and $F_{1}\;score$ (lower half) ± standard deviation. The best performing approach is highlighted in grey. Moreover, a paired student’s *t*-test with a significance level of 5% was performed to check the significance in differences between the accuracies obtained by each pair of approaches for each classification problem. ${}^{1,2,3,4,5,6,7,8,9}$ indicate a significant improvement compared to the HCF, dPhEDA, TVSymp, MTVSymp, “HCF combined”, MLP, CNN, LSTM, and CAE approaches, respectively.

Method		*B* vs. NP	*B* vs. $P_{1}$	*B* vs. $P_{2}$	*B* vs. $P_{3}$	*B* vs. $P_{4}$
	HCF	51.61 ± 12.51 ^2,3,4,5,7,8,9^	$56.11\pm 11.19{\;}^{3,4,8}$	$61.29\pm 12.35{\;}^{3,4}$	73.99 ± 13.02 ^2,3,4,5,7,8,9^	$87.21\pm 11.32{\;}^{2,3,4,5}$
	dPhEDA	49.21±11.45	$57.19\pm 12.41{\;}^{3,4,8}$	$63.18\pm 12.47{\;}^{1,3,4,8}$	$70.36\pm 12.58{\;}^{3,4}$	$85.27\pm 12.02{\;}^{3,4}$
	TVSymp	$48.80\pm 13.85{\;}^{5}$	51.23±12.65	57.46±12.96	66.83±13.57	80.44±13.53
	MTVSymp	48.32±12.15	51.15±12.21	57.41±13.63	67.45±14.15	79.90±14.40
Acc	HCF combined	47.00±12.40	$57.38\pm 09.76{\;}^{3,4,8}$	$64.28\pm 13.10{\;}^{1,2,3,4,6,8}$	$72.45\pm 13.13{\;}^{2,3,4,8}$	$86.34\pm 12.27{\;}^{2,3,4}$
	MLP	$50.43\pm 12.92{\;}^{2,4,5}$	$57.55\pm 11.17{\;}^{3,4,8}$	$63.17\pm 12.63{\;}^{1,3,4,8}$	$72.84\pm 14.27{\;}^{2,3,4}$	$86.50\pm 12.76{\;}^{2,3,4}$
	CNN	48.82±13.72	58.08 ± 11.08 ^3,4,8^	$64.12\pm 12.16{\;}^{1,2,3,4,8}$	$72.00\pm 13.84{\;}^{2,3,4}$	87.41 ± 11.99 ^2,3,4,5,6^
	LSTM	48.00±12.10	$53.65\pm 11.42{\;}^{3,4}$	$60.82\pm 12.74{\;}^{3,4}$	$71.23\pm 13.00{\;}^{3,4}$	$86.32\pm 12.22{\;}^{2,3,4}$
	CAE	48.92±11.18	$57.07\pm 12.48{\;}^{3,4,8}$	64.75 ± 12.70 ^1,2,3,4,6,8^	$72.60\pm 13.65{\;}^{2,3,4,8}$	$86.88\pm 12.36{\;}^{2,3,4,5}$
	HCF	50.43 ± 12.70	54.38±11.96	59.68±13.10	72.33 ± 14.79	86.67±12.16
	dPhEDA	48.27±11.56	56.04±12.50	61.82±13.12	69.03±13.55	84.71±12.82
	TVSymp	47.96±14.18	50.11±13.06	56.12±13.40	65.69±14.16	79.92±14.10
	MTVSymp	47.45±12.38	49.86±12.76	56.34±14.07	66.40±14.78	79.44±14.84
$F_{1}$	HCF combined	45.86±12.63	55.68 ± 10.47	62.97 ± 13.75	71.02±14.48	85.65±13.51
	MLP	49.31±13.34	56.12±11.49	61.86±13.25	71.57±15.37	85.82±13.98
	CNN	47.54±13.98	56.39 ± 11.62	62.73±12.95	70.51±15.17	86.70 ± 13.41
	LSTM	46.96±12.28	52.07±12.03	59.39±13.50	69.81±14.14	85.70±13.30
	CAE	47.48±11.6	55.35±13.19	63.46 ± 13.46	71.25±14.95	86.30±13.40

**Table 10:** 5× LOSO average performance using the EDA (RespiBan) sensor of the PMDB for several feature extraction methods in combination with a Random Forest. In contrast to previous tables, the CoVAS parameters are used as label here. Performance metrics are given as average accuracy (upper) and $F_{1}\;score$ (lower half) ± standard deviation. The best performing approach is highlighted in grey. Moreover, a paired student’s *t*-test with a significance level of 5% was performed to check the significance in differences between the accuracies obtained by each pair of approaches for each classification problem. ${}^{1,2,3,4,5,6,7,8,9}$ indicate a significant improvement compared to the HCF, dPhEDA, TVSymp, MTVSymp, “HCF combined”, MLP, CNN, LSTM, and CAE approaches, respectively.

Method		$C_{0}$ vs. $C_{1}$	$C_{0}$ vs. $C_{2}$	$C_{0}$ vs. $C_{3}$	$C_{0}$ vs. $C_{4}$
	HCF	$66.57\pm 10.04{\;}^{2,3,4,5,7}$	$83.04\pm 08.78{\;}^{2,3,4,8}$	88.73 ± 08.05 ^2,3,4,7,8,9^	93.78 ± 06.43 ^2,3,4,7,8^
	dPhEDA	$64.70\pm 09.32{\;}^{3,4}$	$81.84\pm 09.84{\;}^{3,4,8}$	$86.87\pm 08.31{\;}^{3,4}$	$92.71\pm 07.33{\;}^{3,4,8}$
	TVSymp	61.42±10.59	78.61±10.43	83.28±08.51	$89.39\pm 07.25{\;}^{4}$
	MTVSymp	61.99±09.17	78.47±10.17	83.50±08.94	88.35±07.88
Acc	HCF combined	$66.04\pm 09.48{\;}^{2,3,4}$	$83.22\pm 09.45{\;}^{2,3,4,8}$	$88.49\pm 07.75{\;}^{2,3,4,8,9}$	$93.78\pm 06.39{\;}^{2,3,4,6,7,8}$
	MLP	66.47 ± 09.39 ^2,3,4,7,9^	$82.66\pm 09.02{\;}^{3,4,8}$	$88.43\pm 07.96{\;}^{2,3,4,8,9}$	$93.22\pm 06.98{\;}^{2,3,4,8}$
	CNN	$65.54\pm 09.84{\;}^{2,3,4}$	$82.83\pm 08.85{\;}^{2,3,4,8}$	$87.94\pm 07.90{\;}^{2,3,4}$	$93.05\pm 06.63{\;}^{3,4,8}$
	LSTM	$65.85\pm 09.25{\;}^{3,4}$	$81.52\pm 09.46{\;}^{3,4}$	$87.65\pm 08.71{\;}^{2,3,4}$	$92.52\pm 07.35{\;}^{3,4}$
	CAE	$66.15\pm 09.49{\;}^{2,3,4,7}$	83.10 ± 08.91 ^2,3,4,8^	$87.40\pm 09.36{\;}^{2,3,4}$	$93.50\pm 06.83{\;}^{2,3,4,8}$
	HCF	57.75±12.11	68.25±16.37	79.09 ± 15.53	87.60 ± 13.48
	dPhEDA	56.69±10.18	65.81±16.31	74.61±16.36	85.12±16.16
	TVSymp	54.69±10.35	62.75±15.27	70.60±14.70	81.05±12.54
	MTVSymp	55.31±09.75	62.52±15.05	71.11±13.94	79.53±12.78
$F_{1}$	HCF combined	58.26±11.40	68.19±17.00	78.57±15.57	87.52±14.27
	MLP	58.54 ± 10.84	68.32±16.09	78.35±16.11	87.43±13.64
	CNN	57.61±10.89	68.41±15.95	77.87±15.78	86.44±14.52
	LSTM	57.99±10.22	66.91±15.94	77.55±16.54	85.52±15.12
	CAE	58.27±10.90	68.62 ± 15.97	77.28±17.03	87.29±14.18

## Data Availability

The BVDB is available under http://www.iikt.ovgu.de/BioVid.html (accessed on 15 July 2021). Further data sharing is not applicable to this article.

## Abbreviations

The following abbreviations are used in this manuscript:

**PMDB**	PainMonit Database
**BVDB**	BioVid Heat Pain Database
**BVP**	Blood Volume Pulse
**ECG**	Electrocardiogram
**GSR**	Galvanic Skin Response
**sEMG**	surface Electromyogram
**EMG**	Electromyogram
**HR**	Heart Rate
**EDA**	Electrodermal Activity
**GSR**	Galvanic Skin Response
**SCL**	Skin Conductance Level
**SCR**	Skin Conductance Respons
**IBI**	Inter-Beats-Interval
**ACC**	Accelerometer
**RB**	respiBAN Professional
**E4**	Empatica E4
**RF**	Random Forest
**SVM**	Support Vector Machine
**HCF**	Hand-Crafted Features
**MLP**	Multi-Layer Perceptron
**NN**	Neural Network
**CNN**	Convolutional Neural Network
**AE**	Autoencoder
**CAE**	Convolutional Autoencoder
**LSTM**	Long Short-Term Memory
**RNN**	Recurrent Neural Network
**DDCAE**	Deep Denoising Convolutional Autoencoder
**ReLU**	Rectified Linear Unit
**ADAM**	Adaptive Moment estimation
**LOSO**	Leave-one-subject-out
**CV**	Cross Validation
**RMSE**	Root Mean Square Error
**MSE**	Mean Squared Error
**NRS**	Numerical Rating Scale
**VAS**	Visual Analogue Scale
**CoVAS**	Computerised Visual Analogue Scale
**IASP**	International Association for the Study of Pain
**STD**	Standard Deviation
**VAR**	Variance
**cvxEDA**	convex EDA optimisation method
**dPhEDA**	derivative of phasic component of EDA
**TVSymp**	spectral features time-varying index of sympathetic activity
**MTVSymp**	modified spectral features time-varying index of sympathetic activity

## Appendix A

### Appendix Comparison to the Cnn Architecture Published by Thiam et al.

While there are not many deep learning architectures including all implementation steps to compare results proposed in the past, Thiam et al. [30] provided the needed information to reproduce their approach in their paper. Thus, we implemented their CNN network (later referred to as “Thiam”) and compared it to ours (still referred to as “CNN”). Although relying on more layers, Thiam et al. network performs worse compared to ours using the proposed feature extraction framework. Table A1 and Table A2 show the average results of a 5 × LOSO scheme for the BVDB and PMDB, respectively. For the PMDB the heater label is used to be comparable to the BVDB that contains only temperature labels and thus Thiam et al. is trained on it exclusively.

**Table A1:** 5× LOSO average performance using the EDA sensor of the BVDB for the Thiam and CNN architectures. Performance metrics are given as average accuracy (upper) and $F_{1}\;score$ (lower half) ± average standard deviation of each individual LOSO run. Moreover, the best performing approach is highlighted in grey.

Method		$T_{0}$ vs. $T_{1}$	$T_{0}$ vs. $T_{2}$	$T_{0}$ vs. $T_{3}$	$T_{0}$ vs. $T_{4}$
Acc	CNN	57.08±11.70	64.05 ± 15.07	72.94 ± 13.96	82.93 ± 14.22
	Thiam	57.89 ± 12.61	64.01±14.40	72.78±15.14	81.98±15.37
$F_{1}$	CNN	56.44±12.01	62.93±15.92	72.15±14.77	82.52±14.86
	Thiam	56.97 ± 13.06	62.94 ± 15.21	71.93±16.00	81.58±15.96

**Table A2:** 5× LOSO average performance using the EDA (RespiBan) sensor of the PMDB for the Thiam and CNN architectures. Performance metrics are given as average accuracy (upper) and $F_{1}\;score$ (lower half) ± standard deviation. Moreover, the best performing approach is highlighted in grey.

Method		*B* vs. NP	*B* vs. $P_{1}$	*B* vs. $P_{2}$	*B* vs. $P_{3}$	*B* vs. $P_{4}$
Acc	CNN	48.82±13.72	58.08 ± 11.08	64.12 ± 12.16	72.00±13.84	87.41 ± 11.99
	Thiam	49.71 ± 11.58	54.37±11.69	62.37±11.59	72.88 ± 12.12±	86.52±12.13
$F_{1}$	CNN	47.54±13.98	56.39 ± 11.62	62.73 ± 12.95	70.51±15.17	86.70 ± 13.41
	Thiam	48.41 ± 11.89	52.69±12.40	61.10±12.37	71.41 ± 15.97	85.80±13.48

Although yielding good results for no pain vs. low pain, Thiam’s network does not perform better for tasks no pain vs. high pain ($T_{0}$ vs. $T_{4}$/*B* vs. $P_{4}$). Because of the obtained results and the higher complexity of Thiam’s network, it was not used in our experiments. Moreover, there is a discrepancy between accuracy results reported in Thiam et al. [30] and the ones yielded in our setup. Differences can be argued by variations in the pre-processing chain and unequal evaluation scheme using a single LOSO scheme and a 5 × LOSO average in [30] and our framework, respectively.
